# Comparative Studies of the Influence of Relative Humidity and Temperature on the Longevity and Fecundity of the Parasitoid, *Cotesia flavipes*

**DOI:** 10.1673/031.007.1901

**Published:** 2007-04-09

**Authors:** Emana GD

**Affiliations:** Department of Biology, Addis Ababa University, P.O.Box 1176, Addis Ababa, Ethiopia

**Keywords:** augmentative release, *Chilo partellus*, biological control

## Abstract

The parasitoid, *Cotesia flavipes* (Cameron) (Hymenoptera: Braconidae), was introduced for biological control of the stemborer, *Chilo partellus* (Swinhoe) (Lepidoptera: Crambidae), in eastern and southern African countries. The parasitoid became firmly established in Ethiopia, with varying density and distribution in various regions of the country indicating that there are factors regulating the success of the parasitoid. From previous studies, it was known that the population of the parasitoid released, the type of host, and temperature highly affect some of the biological parameters of the parasitoid. The current studies were undertaken to understand the individual and interactive effect of temperature and relative humidity on the longevity and fecundity of *C. flavipes.* The study was conducted on *C. flavipes* collected from the Melkassa Agricultural Research Center Experimental Field, Ethiopia. *C. flavipes* was reared in the laboratory on *C. partellus* feeding on pieces of sorghum stem. The longevity experiment was conducted at 10, 20, 30 and 40 °C, while the fecundity experiment was conducted at 20, 25, 28 and 30 °C. For both experiments 40–50%, 60–70% and 80–90% relative humidity regimes were used. The results obtained indicate that the interactive effect of temperature and relative humidity significantly affected the longevity, the number of oocytes, and fecundity of *C. flavipes* implying that the two factors play an important role in the success of the parasitoid as a biocontrol agent against *C. partellus.* The results obtained suggests the importance of the selection of target release sites for maximum efficiency of the parasitoid, which can have a positive impact on the on-going augmentative release of *C. flavipes* in Ethiopia.

## Introduction

*Cotesia flavipes* (Cameron) (Hymenoptera: Braconidae) is a gregarious, larval endo-parasitoid of gramineae stemborers from the Indo-Australian region. In its aboriginal home, *C. flavipes* attacks *Chilo partellus* (Swinhoe) (Lepidoptera: Crambidae) and other Asian stemborers. *Cotesia flavipes* was used in three attempts to achieve classical biological control of *C. partellus* in Africa (Overholt et al., 1994a; Overholt et al., 1997). However, it was only the last attempt by the International Centre of Insect Physiology and Ecology (ICIPE) in 1993 that reported successful establishment in different eastern and southern African countries where parasitoids were field released. A baseline survey was done in 1999 to describe the species composition, distribution and the status of cereal stemborers and their natural enemies in Ethiopia. *C. flavipes* was discovered to be already present in Ethiopia (Emana et al., 2002; Emana et al., 2003 a, b; Emana et al., 2004). The source of *C. flavipes* population in Ethiopia is not known, but the most likely source could be the east African releases by ICIPE such as Somalia, Kenya, Tanzania and Uganda. A Polymerase Chain Reaction- Restricted Fragment Length Polymorphism (PCR-RFLP) analysis indicated that there was no difference among *C. flavipes* population from Ethiopia, Kenya, Tanzania, Uganda, India, North Pakistan and South Pakistan (Emana unpublished, 2006). Gene sequencing of the different *C. flavipes* populations mentioned above is underway to quantify the genetic similarities.

Since its first record in Ethiopia, *C. flavipes* has increased its parasitism rate from 7.5% in 1999 to 58.8% in 2005 (Emana, unpublished data). The rate of parasitism varies from region to region in Ethiopia. Within-region variation is also large, clearly indicating that there are biotic and abiotic factors regulating the biological activities of the parasitoid such as longevity and fecundity. Emana et al. (2003, 2004) demonstrated the effect of different temperatures and relative humidities regimes on life table parameters and the developmental period of *C. flavipes* suggesting that the biological parameters of the parasitoid are highly affected by individual factors and factors interaction. Ngi-Song et al. (1995), Mbapila (1997), Potting (1997) and Jiang et al. (2004) reported differential performance of *C. flavipes* on different stemborers reared under different temperature regimes. South Wood (1978) indicated that longevity and fecundity are among the most important biological parameters for measuring the fitness of biological control agents for biological control programs.

Though *C. flavipes* is doing well in Ethiopia, grain losses due to *C. partellus* are still large which warrant effective management of the pest through integrated management. The integrated management program of *C. partellus* in Ethiopia mainly focuses on augmentative release of *C. flavipes* cocoons (approximately 5000 individuals per location) reared in Ethiopia. Conservation of parasitoids, mainly by minimizing pesticide use, and other control measures such as cultural control are also used. Hence, the current experiment was conducted to determine the longevity and fecundity of *C. flavipes* collected from Melkassa Agricultural Research Center (MARC) experimental field in central Ethiopia in order to optimize the augmentative release of *C. flavipes* in Ethiopia for the control of *C. partellus.* The Ethiopian population of *C. flavipes* was used for these experiments as the previous work by Emana (2002) indicated that populations of *C. flavipes* from India and Pakistan showed differences in longevity and fecundity.

## Materials and Methods

Colonies were started from one cocoon of *C. flavipes* that was randomly selected from 12 cocoons collected from the MARC experimental field, and were maintained on laboratory reared fourth instar *C. partellus* larvae at MARC Entomology Laboratory. Larvae of *C. partellus* were reared in the laboratory on its natural diet of soft sorghum leaf sheaths for first and second larval instars and 3–5 cm long sorghum stems for third to sixth larval instars. Incubators were set at constant temperatures of 10, 20, 30 and 40 °C for the longevity study and at 20, 25, 28 and 30 °C for the fecundity study. Different salt solutions were used (see below) to maintain the relative humidity at 40–50, 60–70 and 80–90% for both studies (Emana et al., 2003, 2004). In each incubator three 0.35 × 0.35 × 0.35 m insect cages were placed, each one was maintained at one of the 3-humidity regimes. In each insect cage a humidity gauge was placed to check the accuracy of the humidity treatments. If the humidity deviated, adjustments were made.

A container of 2 kg of Zn(NO3)2.2H2O was used to maintain relative humidity at 40–50% at the temperatures of 20–40°C. The cages were not sealed. For 10°C cages were sealed with vasoline and a container of 2–3 kg of CaC12 was used to maintain the relative humidity at 40–50%. To maintain the relative humidity at 60–70% and at 20–40°C, a container of 0.5 kg of Ca(NO3)2.4H2O and another with water in a sealed cage were used. To maintain the same relative humidity at 10°C, a container of 4 kg of Zn(NO3)2.6H2O was used. For the highest relative humidity, 80–90%, a container of 0.5 kg KCl was used for all temperature regimes, but a container of water was also present at the higher temperatures of 20, 30 and 40°C. The experiments were designed in a completely randomized design in a factorial arrangement. The experiments were replicated 30 times for longevity, 10 times for potential fecundity (oocyte number) and 20 times for realized fecundity. Each replication consisted of one insect.

**Table 1.**
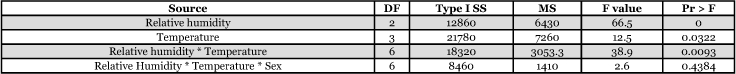
ANOVA for *Cotesia flavipes* longevity

### Longevity

Cocoons of *C. flavipes* were held at 28 °C until parasitoid emergence. Immediately after emergence, they were allowed to mate for 12 hours then placed individually in 2.5 × 7 cm glass vial with a drop of 20% honey/water solution to serve the adult as a food. Vials were then placed in incubators set at the various respective temperature and relative humidity regimes. Mortality was recorded twice a day until all parasitoids were dead.

### Realized fecundity

Twelve-hour-old mated females of *C. flavipes* were held under light for 30 minutes to activate them for oviposition. Each female was then offered a fourth instar larva of *C. partellus* as a host. The parasitized larvae were placed individually in Petri dishes with a piece of sorghum stem disinfected with sodium hypochlorite (0.05). Each parasitized larva was randomly assigned to a treatment regime. Every three days, fresh sorghum stems were provided to the larvae until cocoon formation, pupation and/or death of the larva. Daily observation continued until the emergence of the adult wasps. After emergence, the adults were allowed to mate for 12 hours.

**Table 2.**
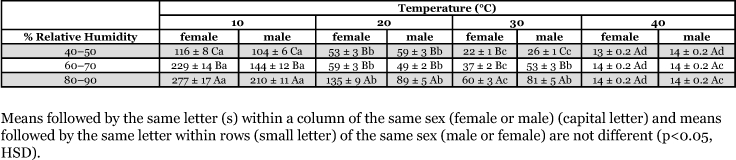
Comparison of *C. flavipes* (mean hours ± SD) at different levels of relative humidity and temperatures for longevity of adult females and males.

### Oocyte number

From the batches of each treatment regime, 10 female wasps were randomly taken and dissected. The ovaries of each female were removed, mounted on slides and the oocytes were counted under a microscope.

### Fecundity

From the batches of each treatment regime, 20 adult females were randomly selected and provided with a fourth instar *C. partellus* larva. The parasitized larva was placed in a Petri dish, provided with a sorghum stem, and placed back in the treatment regime. Each female wasp was provided with a *C. partellus* larva twice a day until her death according to the established protocol by Emana et al. (2004). The number of female and male progeny were recorded upon emergence. After formation of wasp cocoons, the host larva was dissected and the parasitoid cocoons, and larvae which had failed to develop into cocoons, were counted. These were summed per day per female to obtain a measure of realized fecundity. Replicates in which the wasp progeny were all males, or the host larva pupated or died, were excluded from the analysis.

**Table 3.**
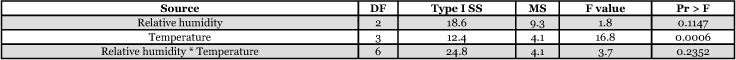
ANOVA for *Cotesia flavipes* Oocyte number

## Data analysis

Data were analyzed by SAS computer software (SAS Institute, 1988) using the General Linear Model (GLM). Two-way analyses using Proc Sort command was performed where the factors interactions were significant. Data were transformed by logarithmic transformation for analysis and back transformed for reporting (Gomez and Gomez, 1984). Significant means were separated using Tukey's studentized range test (HSD).

## Results

### Longevity

The longevity experiment indicated that temperature, relative humidity, and their interactions, significantly affected the survival period of the adult wasps. The interaction of temperature, relative humidity and sex did not significantly affect longevity (Table 1).

### Effect of relative humidity on longevity

Adult *C. flavipes* lived significantly longer at the highest relative humidity (80–90%) at the temperatures of 10, 20 and 30 °C. Relative humidity had no effect on the longevity of wasps at 40 °C. The longevity of the adult wasps was significantly longer at 60–70% and 80–90% relative humidities, particularly at lower temperatures (Table 2).

### Effect of temperature on longevity

As temperature increases from 10 to 40 °C the survival time of *C. flavipes* significantly shortened. The longevity of the female *C. flavipes* adult was significantly higher at 10 and 20 °C (Table 2).

**Table 4.**
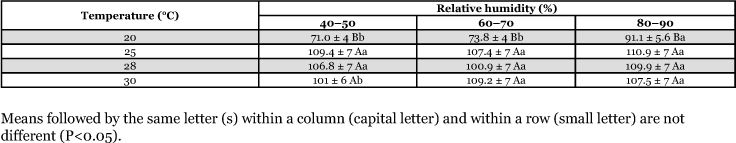
Effect of temperature and relative humidity on the number of oocytes of *C. flavipes* (means ± SD)

### Oocyte number

Oocyte number was not significantly affected by relative humidity, but was significantly affected by temperature. The interaction of relative humidity and temperature was not significant (Table 3).

### Effect of relative humidity on oocyte number

Significantly higher oocyte numbers were recorded at 80–90% relative humidity. Relative humidity did not significantly affect the number of oocytes at 25 and 28 °C (Table 4).

### Effect of temperature on oocyte number

Significantly lower oocyte numbers were recorded at lower temperature (20 °C), but no significant differences were observed among the temperatures of 25, 28 and 30 °C (Table 4).

### Realized fecundity

Realized fecundity was significantly affected by temperature and relative humidity, but their interaction was not significant (Table 5).

### Effect of relative humidity on realized fecundity

Significantly higher realized fecundity was recorded at 80–90% relative humidity. At 25 and 28 °C, relative humidity had no effect on realized fecundity (Table 6).

### Effect of temperature on realized fecundity

A significant effect of temperature on realized fecundity of *C. flavipes* was seen at the lowest and intermediate relative humidities. However, the lowest fecundity was seen at 20 °C at the lowest relative humidity, but at 30 °C at the intermediate relative humidity (Table 6).

**Table 5.**
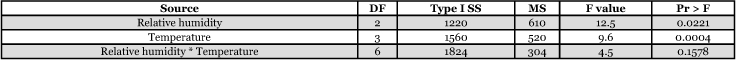
ANOVA for *Cotesia flavipes* fecundity

## Discussion

The effects of temperature on life history parameters of insects such as longevity and fecundity (Mbapila, 1997; Rahim et al., 1991) have been intensively studied. Very few studies have been done on the effect of temperature and relative humidity on these life history parameters (Yadav and Chaudhary, 1987; Ali et al., 1991; Rahim et al., 1991; Emana et al., 2003 and 2004). Emana (2002) reported differences in longevity and fecundity in India and Pakistan populations of *C. flavipes* at different temperatures and relative humidities. The present study examines these parameters for an Ethiopian population of the parasitoid. As the source of *C. flavipes* reported from Ethiopia is not known, obtaining some life history parameters under different temperatures and relative humidity is important. Ethiopia has a wide range of climates ranging from dry lowland to the wet highland where cereals such as maize and sorghum grow abundantly. *C. partellus* was reported as a pest of sorghum and maize up to 2080 meters above sea level (Emana et al., 2004). The level of parasitism of *C. partellus* by C. *flavipes* under field conditions varies by elevation (Emana, unpublished data). The temperature and relative humidity of locations at different elevations are significantly different, which may result in differential parasitism by affecting biological parameters such as longevity and fecundity of *C. flavipes.* The abundance of *C. partellus* and a suitable climate are the most important factors attributed to the success of *C. flavipes* in Ethiopia.

### Longevity

The present findings showed that relative humidity, temperature and their interaction significantly affected the longevity of *C. flavipes*, which suggests that these physical factors are important components in determining the establishment of *C. flavipes.* As *C. flavipes* adults live for only a few days (Mohyuddin and Greathead, 1970; Overholt et al., 1997; Potting, 1997; Wiedenmann et al., 1992) during which mating and egg laying must take place, (Potting, 1996; Van Den Bosch et al., 1979) longevity can severely affect fecundity. As far as *C. flavipes* is concerned, longevity may not affect the mating as it occurs immediately after emergence (Overholt et al., 1994 a and b). However, longevity has a great impact on egg laying. Adult female wasps can have about 100 eggs in her ovary after mating. At least half of these eggs are usually laid before the death of the adult female. This can only happen if physical factors such as temperature and relative humidity, among other factors, are optimum to the parasitoid.

**Table 6.**
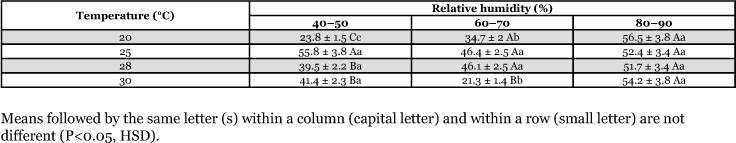
Effect of temperature and relative humidity on fecundity of *C. flavipes* (Mean number of progeny ± SD)

The current longevity experiment, shows that *C. flavipes* only survive for half a day at the highest temperature regardless of relative humidity, which may not be sufficient time for the female parasitoid to lay her eggs before dying. Although, *C. partellus* is expanding into higher elevations (up to 2080 m), it is mainly found in lowland areas, which have an average daily temperature of above 28 °C, implying that *C. flavipes* must be effective at higher temperatures. These results can be useful for determining the stage of the parasitoid to be released for biological control of *C. partellus.* Huffker and Rabb (1984) indicated that the stage of the parasitoid released significantly affected the establishment of the parasitoid. Especially when the release site is far away from the laboratory where the parasitoid is reared, it is important to use the cocoon stage for release. The longer longevity of *C. flavipes* at lower temperature (10 and 20 °C) may not be of high value as the target pest is not present or is not a problem in areas having such low temperatures. Thus, the survival of the parasitoid under such low temperature may be determined by the availability of the host. In the current study, the adult female of *C. flavipes* lived significantly longer than the male at some temperature and relative humidity regimes, which agrees with the results of Mbapila (1997). In general, for parasitoids like *C. flavipes* that have a short life span, knowing their longevity under different conditions not only helps to optimize the release strategy, but also helps to decide which stage of the parasitoid should be used for field release. Based on the result of the current longevity experiment, the cocoon stage of *C. flavipes* should be considered for field release for locations having a daily mean temperature above 20 °C for releases in locations far away from the rearing site.

### Fecundity

Pests and parasites, such as parasitoids, are population dependent, i.e., high populations lead to high infestation and high parasitism, both of which depend on fecundity (South Wood, 1978; Odum 1983; Clark et al., 1967; Muegge and Lambdin, 1989; Turnock and Muldrew, 1971). Fecundity is affected by a number of biotic and abiotic factors. Yadav and Chaudhary (1987) and Ali et al. (1991) reported that temperature, relative humidity and their interaction highly governs the efficiency of a given parasitoid, mainly by affecting the fecundity of the parasitoid. In the current experiments, low temperatures were found to result in reduction of the number of oocytes, and the fecundity of *C. flavipes* was found to be significantly higher when it was reared at temperatures of 25–30 °C, which agree with earlier findings by Emana et al. (2004) who reported the highest intrinsic rate of growth of *C. flavipes* for Indian and Pakistan populations at the same temperature ranges. This clearly indicates that for maximum efficiency, *C. flavipes* release should be done in areas having mean daily average temperature of 25 to 30 °C, which are normally characteristic of lowland areas where *C. partellus* is mainly found. The current study indicates that none of the treatment regimes tested showed deleterious effects on the fecundity of *C. flavipes.* However, the interaction of the factors at different levels resulted in variations in fecundity, suggesting that differential establishment rates of *C. flavipes* depend on temperature and relative humidity. The release of *C. flavipes* is a continuous process in *C. partellus* prone areas in Africa in general, and Ethiopia in particular, as the level of parasitism varies considerably from location to location. Hence, redistribution of the parasitoid within the country by mass rearing in the laboratory and releasing in the field (augmentative release) is mandatory for effective management of *C. partellus.*

### Conclusion and Recommendation

The results obtained in this study strongly support the predictive approach of classical biological control whereby extensive prerelease studies should be conducted before the release of biological control agents. In this study, under controlled laboratory conditions temperatures ranging from 25–28 °C and relative humidity of 60–70% were found to be the best combination for raising *C. flavipes.* Environmental conditions for field release should approximate a temperature range of 20–30 °C and relative humidity of 40–
